# Serum lipid metabolism characteristics and potential biomarkers in patients with unilateral sudden sensorineural hearing loss

**DOI:** 10.1186/s12944-024-02189-8

**Published:** 2024-06-29

**Authors:** Xiaoyan Chen, Zhong Zheng, Daoyu Xie, Liang Xia, Yi Chen, Hongjun Dong, Yanmei Feng

**Affiliations:** 1 Department of Otolaryngology-Head and Neck Surgery, Shanghai Jiao Tong University School of Medicine Affiliated Sixth People’s Hospital, 600 Yishan Road, Xuhui District, Shanghai, China; 2 Shanghai Key Laboratory of Sleep Disordered Breathing, Shanghai, China; 3 Department of Otolaryngology Head and Neck Surgery, Xijing Hospital, Fourth Military Medical University, Xi’an, Shaanxi Province China; 4 Department of Otolaryngology-Head and Neck Surgery, Affiliated Hospital of Hangzhou Normal University, Hangzhou, Zhejiang Province China; 5 Department of Otolaryngology-Head and Neck Surgery, Zhangjiagang TCM Hospital, Affiliated to Nanjing University of Chinese Medicine, Zhangjiagang, Jiangsu Province China

**Keywords:** Sudden sensorineural hearing loss, Lipidomics, Risk factors, Biomarkers

## Abstract

**Background:**

Glycerophospholipids (GPLs) are essential for cell membrane structure and function. Sphingomyelin and its metabolites regulate cell growth, apoptosis, and stress responses. This study aimed to investigate lipid metabolism in patients experiencing sudden sensorineural hearing loss across all frequencies (AF-SSNHL).

**Methods:**

The study included 60 patients diagnosed with unilateral AF-SSNHL, among whom 30 patients had a level of hearing improvement ≥ 15 dB after 6 months of follow-up. A propensity score-matched (2:1) control group was used. Liquid chromatography‒mass spectrometry based untargeted lipidomics analysis combined with multivariate statistics was performed to investigate the lipids change. The “lipidome” R package and weighted gene co-expression network analysis (WGCNA) were utilised to assess the lipids’ structural features and the association between lipids and hearing.

**Results:**

Lipidomics successfully differentiated the AF-SSNHL group from the control group, identifying 17 risk factors, mainly including phosphatidylcholine (PC), phosphatidylethanolamine (PE), and related metabolites. The ratios of lysophosphatidylcholine/PC, lysophosphatidylethanolamine/PE, and lysodimethylphosphatidylethanolamine/PE were upregulated, while some glycerophospholipid (GPL)-plasmalogens were downregulated in the AF-SSNHL group, indicating abnormal metabolism of GPLs. Trihexosylceramide (d34:1), PE (18:1e_22:5), and sphingomyelin (d40:3) were significantly different between responders and nonresponders, and positively correlated with hearing improvement. Additionally, the results of the WGCNA also suggested that partial GPL-plasmalogens were positively associated with hearing improvement.

**Conclusion:**

AF-SSNHL patients exhibited abnormally high blood lipids and pronounced GPLs metabolic abnormalities. Sphingolipids and GPL-plasmalogens had an association with the level of hearing improvement. By understanding the lipid changes, clinicians may be able to predict the prognosis of hearing recovery and personalize treatment approaches.

**Supplementary Information:**

The online version contains supplementary material available at 10.1186/s12944-024-02189-8.

## Background

Sudden sensorineural hearing loss (SSNHL) involves an abrupt sensorineural hearing loss ≥ 30 dB affecting more across a minimum of three sequential frequencies within three days [1]. Some patients fail to recover from hearing loss and experience concomitant tinnitus and vertigo, which may exacerbate the psychological and functional burden [1]. Inflammatory response, viral infection, cochlear microcirculation disturbance, and autoimmunity may be the causes of SSNHL [2]. Vasospasm, stria vascularis dysfunction, and vascular embolism are considered to be the pathogenic factors for SSNHL with flat type and total deafness [3].

A meta-analysis in 2023 found that total cholesterol (TC) levels were considerably elevated in patients experiencing SSNHL when compared to those without the condition [4]. Moreover, a historical cohort research conducted in Taiwan found that the cohort with hyperlipidemia had a 1.62-fold increased risk of developing SSNHL, with a hazard ratio of 1.60 times after adjusting confounding factors [5]. The potential mechanisms underlying the impairment of inner ear microcirculation due to lipid abnormalities include (1) increased blood viscosity, decreased blood flow, and oxygen delivery; (2) activation of the fibrinolytic system, resulting in microvascular embolism; (3) changes in the stria vascularis or hair cell ultrastructure; and (4) inhibition of vasodilator factors, leading to generates sustained capillary constriction [6, 7]. In recent decades, lipidomic techniques have been increasingly utilized to study diseases such as atherosclerosis [8], stroke [9], and myocardial infarction [10], providing a novel approach to explore pathogenesis and identify biomarkers.

Traditional risk factors did not fully account for the incidence risk of cardiovascular diseases, and individuals who met standard criteria through conventional interventions for traditional risk factors still faced a certain degree of risk for the incidence and mortality of cardiovascular diseases [11]. Lipidomics is highly sensitive to changes in lipid molecules and can identify potential metabolic aberrations in the early stages of disease. Biomarkers identified through lipidomic profiling are a significant complement to traditional risk factors. Cochlear microcirculation dysfunction was one of the pathogenic mechanisms of all frequencies SSNHL (AF-SSNHL), and hyperlipidemia had been considered a risk factor for SSNHL in previous studies. Based on this, this study for the first time utilized untargeted lipidomics to investigate lipid metabolism in patients with AF-SSNHL and identified novel biomarkers that may aid in clinical diagnosis and prognosis.

## Methods

### Study population

Eligibility for participation was granted to individuals who sought medical attention within 2 weeks following the onset of sudden hearing loss, as reported by them, and who exhibited a hearing loss of ≥ 30 dB across the frequencies of 0.25, 0.5, 1, 2, 3, 4, and 8 kHz. Recruitment was open from February 2022 to March 2023. Those in the control group completed audiological testing and otolaryngological exams throughout the same time period without experiencing any problems. Excluding criteria for all participants were: (1) atrial fibrillation, heart failure, cerebrovascular diseases, diabetes, malignant tumors, liver or kidney dysfunction, autoimmune disease, thyroid dysfunction, infectious diseases, and malnutrition; (2) Meniere’s disease, vestibular migraine, ear surgery, conductive hearing loss, and other sensorineural hearing loss; (3) pregnant or nursing; (4) under 18 years old; (5) binaural or previous SSNHL; (6) took medication prior to enrollment; (7) lost to follow-up. Ethics approval was granted by the Committee of Shanghai Sixth People’s Hospital (2018-KY-036(K), dated July 24, 2018), adhering to the Helsinki Declaration. Informed permission was gotten from all participants.

### Treatment and evaluation

All patients received systemic glucocorticoid therapy (dexamethasone, 10 mg/day intravenously for 3–5 days, dose progressively reduced for at least 1 week). Then, 4–5 doses of retroaural injections (dexamethasone, 5 mg every other day) were administered. According to Chinese guidelines, batroxobin, neurotrophic factor (mecobalamine) and a microcirculation improving drug (ginkgo biloba extract) were also adopted [3]. Patients with hypertension and hyperlipidemia received appropriate treatment to manage these conditions.

Prior to treatment and six months after treatment, audiometric testing was performed to measure both air and bone conduction thresholds across frequencies of 0.25–8 kHz. Hearing loss was calculated by averaging hearing levels at the impaired frequencies ranging from 0.25 to 8 kHz. The level of hearing improvement was determined by subtracting the post-treatment hearing loss from the pre-treatment hearing loss. Based on hearing improvement, patients were divided into groups of responders as follows: (1) complete recovery (hearing levels reverted to normal [< 20 dB], matching the unimpaired ear or the state before disease onset); (2) partial recovery (hearing improvement exceeding 30 dB); and (3) slight recovery (hearing improvement within 15–30 dB) and non-responders (hearing improvement less than 15 dB) [3].

### Samples and data collection

Baseline data were recorded, including age, gender, medical history, otolaryngological symptoms, blood biochemical test results, and inner ear magnetic resonance imaging. A 5 mL sample of fasting peripheral venous blood was collected using an evacuated blood collection tube without an anticoagulant before receiving treatment. The samples were separated by centrifuging them at 3,000 × g for 10 min. The serum was then frozen, sub-packaged, and kept at-80 °C for untargeted lipidomics (supplementary material 1).

### Data processing and statistical analyses

Stability was assessed via principal component analysis, and characteristic peaks with a relative standard deviation of < 30% were reserved in quality control samples. LipidSearch software (Thermo Fisher Scientific) was used for lipid annotation, peak alignment and filtering utilizing a baseline retention time setting of 0.25 and an m-Score cutoff point of 3. A quantitative list was obtained, including lipids, mass-to-charge ratios (m/z), retention times, grade, and peak area. Sum peak normalization was used to correct the quantitative values and compared data of different magnitudes. Anionic and cationic modes were then amalgamated, and the “80% rule” was applied to discard ions with a presence (non-zero values) lower than 80% across all groups. Individual missing values were imputed using the smallest observed value.

**Fig. 1:**
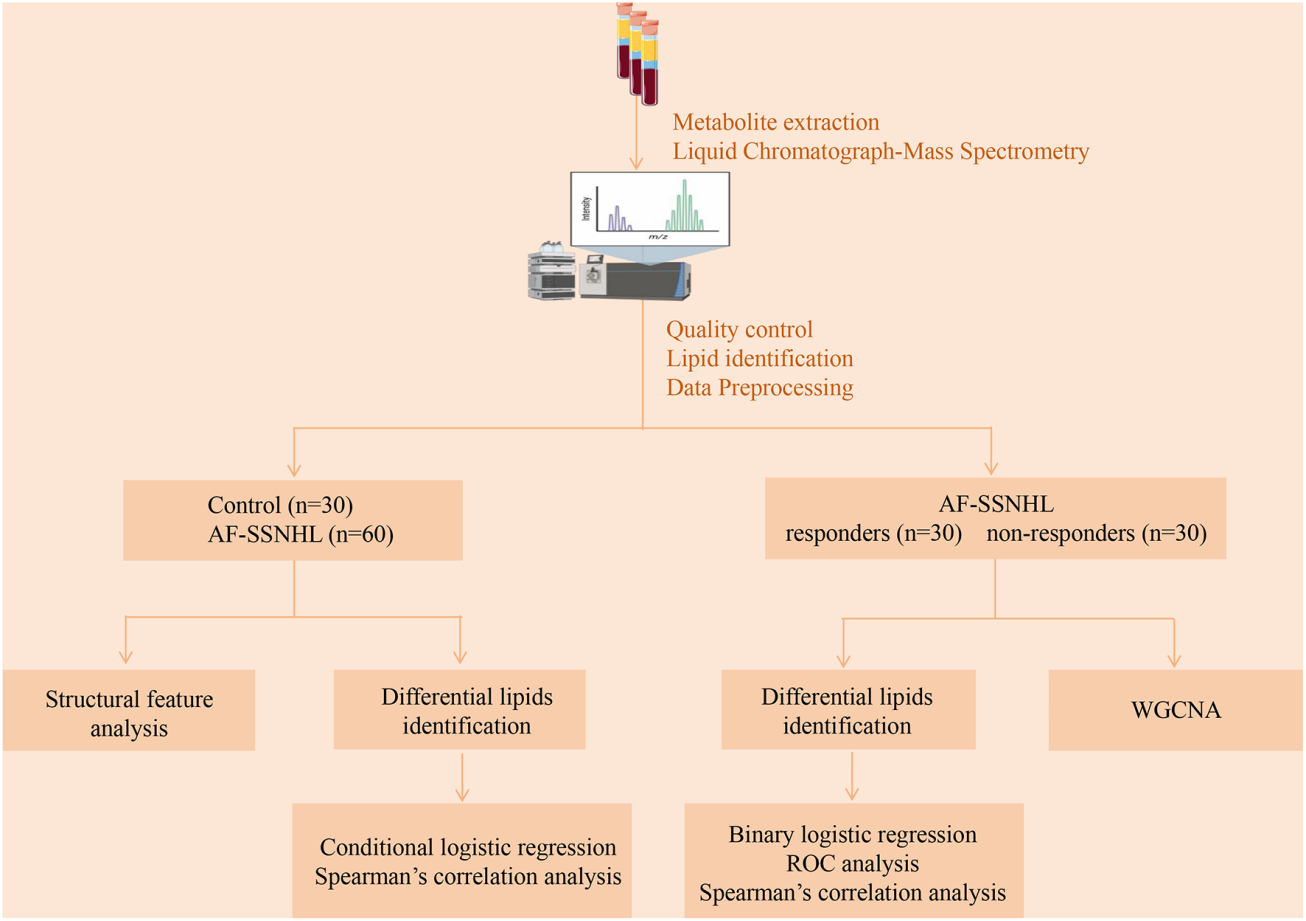
Flow diagram. AF-SSNHL: all frequencies sudden sensorineural hearing loss (AF-SSNHL); WGCNA: weighted gene co-expression network analysis; ROC: receiver operating characteristic curve

SPSS version 26.0 (IBM Corp., Armonk, NY, USA) and R Studio 4.0.4 (R Core Team, Vienna, Austria) were used for the data analysis. Propensity score matching was used to select the control group according to the age, gender and hypertension of AF-SSNHL patients in a ratio of 2:1. Normally distributed variables were presented as means ± standard deviations and subjected to Student’s t-test analysis. Quartiles were used to represent non-normally distributed variables, and the Mann-Whitney U test was used to assess them. Categorical variables were represented as percentages and subjected to chi-square testing. The ratio of the mean was applied to compute fold change (FC). Prior to orthogonal partial least squares discriminant analysis (OPLS-DA), variables were transformed via Log2 and Z-score normalization. The OPLS-DA method was utilized for dimensionality reduction to construct a model elucidating the relationship between lipid profiles and sample groups [12]. The permutation test evaluated whether the indicators of the actual model were significantly higher than the random expected value, preventing overfitting and false-positive results. Variable importance in projection (VIP) scores were leveraged to determine differentially expressed lipids. The screening criteria for significantly differential lipid molecules were *P*-value < 0.05, FC value > 1.5 or ＜ 0.667, and VIP ≥ 1. Bonferroni and false discovery rate-benjamini hochberg (FDR-BH) correction performed on the *P*-values to prevent class I errors. The Log2-transformed data was subjected to both conditional and unconditional logistic regression in order to get the odds ratios (OR) and 95% confidence intervals (CI). Before implementing the logistic regression, the collinearity between all continuous variables was evaluated using the variance inflation factor (< 10). Receiver operating characteristic curve (ROC) analysis was used to evaluate the prediction’s performance. The linear connections were examined using Spearman’s correlation analysis. The utilization of weighted gene co-expression network analysis (WGCNA) facilitated the identification of strongly connected lipid clusters, their interconnection, and their correlation with external phenotypic traits [13]. The “lipidome” R package was used for a structural feature analysis including fatty-acid carbon atoms and double bonds [14]. The main analysis process was shown in Fig. 1.

## Results

### Baseline characteristics

The study included 60 AF-SSNHL patients, among whom 30 patients experienced a hearing improvement of 15 dB or greater following treatment. Compared to the control group, the AF-SSNHL group showed significantly higher levels of TC (*P* < 0.001) and low-density lipoprotein cholesterol (LDL-C) (*P* < 0.001). However, there was no statistically significant difference between the responder and non-responder groups in terms of TC (*P* = 0.293), TG (*P* = 0.154), LDL-C (*P* = 0.229), and HDL-C (*P* = 0.104) (Table 1).

**Table 1:** Baseline data

Variables	Control (*n* = 30)	AF-SSNHL (*n* = 60)	*P*-value	Non-responders (*n* = 30)	Responders (*n* = 30)	*P*-value
Age^a^	46.27 ± 11.25	47.03 ± 13.97	0.795	48.00 (36.25–55.50)	50.00 (34.75–62.25)	0.579
Gender (male %)^c^	16 (53.33%)	31 (51.67%)	0.881	19 (63.33%)	12 (40.00%)	0.071
Hypertension (%)^c^	6 (20.00%)	13 (21.67%)	0.855	21 (70.00%)	26 (86.67%)	0.210
Tinnitus (%)^c^	0 (0)	44 (73.33%)	< 0.001*	9 (30.00%)	7 (23.33%)	0.559
Vertigo (%)^c^	0 (0)	18 (30.00%)	0.001*	20 (66.67%)	22 (73.33%)	0.573
Aural fullness (%)^c^	0 (0)	21 (35.00%)	< 0.001*	21 (70.00%)	18 (60.00%)	0.417
Hearing Loss (dB)^b^	6.34 (5.00–10.00)	79.79 (59.29–89.29)	< 0.001*	83.58 (71.43–91.79)	75.00 (55.72–84.83)	0.013
Time to treatment (days)^b^	/	4.00 (2.00–7.00)	/	5.00 (3.00–8.50)	3.50 (2.00–6.25)	0.050
Hearing improvement (dB)^b^	/	14.50 (0.00–32.14)	/	0.00 (–0.89–3.04)	32.14 (19.08–44.83)	< 0.001*
TC (mmol/l)^a^	4.45 ± 0.52	5.46 ± 1.13	< 0.001*	5.30 ± 1.14	5.61 ± 1.13	0.293
TG (mmol/l)^b^	0.85 (0.65–1.15)	0.96 (0.64–1.48)	0.329	1.01 (0.75–1.54)	0.85 (0.58–1.43)	0.154
HDL-C (mmol/l)^b^	1.33 (1.21–1.51)	1.37 (1.15–1.62)	0.647	1.27 (1.10–1.57)	1.48 (1.27–1.66)	0.104
LDL-C (mmol/l)^a^	2.50 ± 0.51	3.38 ± 1.03	< 0.001*	3.22 ± 0.88	3.54 ± 1.15	0.229

^a^ Means ± standard deviation; ^b^ interquartile range (25–75th ); ^c^ percentages; *P < 0.05; AF-SSNHL: All frequencies sudden sensorineural hearing loss; TC: Total cholesterol; TG: Triglyceride; HDL-C: High-density lipoprotein cholesterol; LDL-C: Low-density lipoprotein cholesterol

### Lipidomic comparisons between the control and AF-SSNHL groups

#### Differential lipids identification

**Fig. 2:**
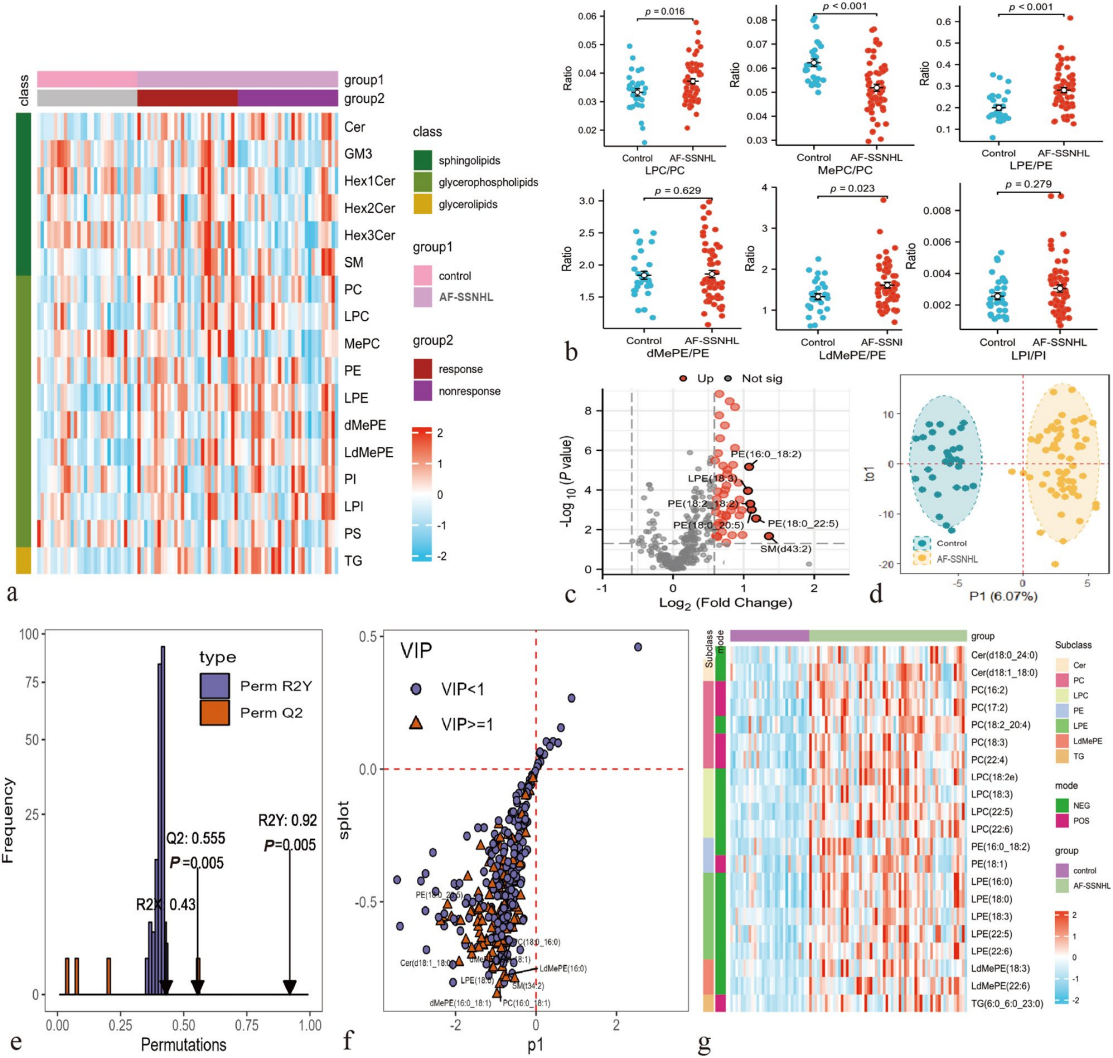
Differential lipids identification. (**a**) Heat map of the lipid subclasses in control vs. all frequencies sudden sensorineural hearing loss (AF-SSNHL), and non-responder vs. responder groups. (**b**) Point diagram of lipid subclass ratio in control vs. AF-SSNHL group. (**c**) Volcano plot of the control vs. AF-SSNHL group. (**d**-**f**) Orthogonal partial least squares discriminant analysis (OPLS-DA): OPLS-DA model (**d**), permutation test (**e**), and s-plot (**f**) of the control vs. AF-SSNHL group. (**g**) Heat map depicting differential lipids in the control vs. AF-SSNHL. Cer: Ceramide, GM3: Monosialoganglioside, Hex1Cer: Monohexosylceramide, Hex2Cer: Dihexosylceramide, Hex3Cer: Trihexosylceramide, SM: Sphingomyelin, PC: Phosphatidylcholine, LPC: Lysophosphatidylcholine, MePC: Methylphosphatidylcholine, PE: Phosphatidylethanolamine, LPE: Lysophosphatidylethanolamine, dMePE: Dimethylphosphatidylethanolamine, LdMePE: Lysodimethylphosphatidylethanolamine, PI: Phosphatidylinositol, LPI: Lysophosphatidylinositol, PS: Phosphatidylserine, TG: Triacylglycerol

331 lipid molecules from glycerolipid (GL), glycerophospholipid (GPL), and sphingolipid (SL) classes were obtained after merging cationic and anionic modes. SLs (*P* = 0.026), GPLs (*P* = 0.009), and GLs (*P* < 0.001) were upregulated in the AF-SSNHL group (Fig. 2a). Additionally, the ratios of lysophosphatidylcholine (LPC)/phosphatidylcholine (PC, *P* = 0.016), methylphosphatidylcholine (MePC)/PC (*P* < 0.001), lysophosphatidylethanolamine (LPE)/ phosphatidylethanolamine (PE, *P* < 0.001), and lysodimethylphosphatidylethanolamine (LdMePE)/PE (*P* = 0.023) were significantly different between the AF-SSNHL group and control group (Fig. 2b).

In total, 159 differential lipid molecules were identified in the control vs. AF-SSNHL (*P* < 0.05), of which 49 lipids met the FC > 1.5 or < 0.67 criteria (Fig. 2c and supplementary material 2). The OPLS-DA analysis demonstrated that the lipid metabolism characteristics of AF-SSNHL group varied from those of control group (Fig. 2d). In permutation test, the R^2^ and Q^2^ values represented the model’s explanatory ability and predictability, respectively. Values of R^2^Y = 0.920, Q^2^ = 0.555, and *P*-value = 0.005 corroborated that the high predictability of the model was not due to randomness or overfitting (Fig. 2e). The VIP values derived from OPLS-DA models served to evaluate the influence and explanatory power of the lipid molecules on group discrimination (Fig. 2f). Finally, 21 differential lipids met the criteria of *P*-value < 1.51e-4 (Bonferroni correction *P*-value = 0.05/331), FC value ≥ 1.5 or ≤ 0.667, VIP ≥ 1 (Fig. 2g).

#### Regression analysis

To ascertain the intensity and direction of the independent variable’s effect on AF-SSNHL, a conditional logistic regression analysis revealed that 17 lipids (PC, PE, and their metabolites) out of 21 differential lipids were risk factors for AF-SSNHL after adjusting for confounding factors, such as age, gender, hypertension, TC, and TG (Table 2).

**Table 2:** Conditional logistic regression model

Variables	OR (95% CI)	*P*-value	Variables	OR (95% CI)	*P*-value
Cer(d18:0_24:0)	1.125 (0.823–1.538)	0.459	PE(16:0_18:2)	1.588 (1.094–2.304)	0.015*
Cer(d18:1_18:0)	1.165 (0.775–1.751)	0.462	PE(18:1)	1.836 (1.118–3.014)	0.016*
PC(16:2)	1.813 (1.120–1.936)	0.015*	LPE(16:0)	2.786 (1.358–5.715)	0.005*
PC(17:2)	2.884 (1.399–5.942)	0.004*	LPE(18:0)	2.477 (1.173–5.233)	0.017*
PC(18:2_20:4)	1.864 (1.178–2.949)	0.008*	LPE(18:3)	1.484 (1.079–2.041)	0.015*
PC(18:3)	1.887 (1.025–3.473)	0.042*	LPE(22:5)	1.475 (0.959–2.270)	0.077
PC(22:4)	2.814 (1.420–5.574)	0.003*	LPE(22:6)	2.299 (1.161–4.552)	0.017*
LPC(18:2e)	2.160 (1.097–4.255)	0.026*	LdMePE(18:3)	1.520 (1.022–2.260)	0.039*
LPC(18:3)	1.556 (1.019–2.377)	0.041*	LdMePE(22:6)	2.005 (1.100–3.653)	0.023*
LPC(22:5)	2.652 (1.416–4.966)	0.002*	TG(6:0_6:0_23:0)	1.396 (0.883–2.205)	0.153
LPC(22:6)	1.986 (1.081–3.650)	0.027*			

OR: Odds Ratio; CI: Confidence Interval; *P < 0.05; Cer: Ceramide; PC: Phosphatidylcholine; LPC: Lysophosphatidylcholine; PE: Phosphatidylethanolamine; LPE: Lysophosphatidylethanolamine; LdMePE: Lysodimethylphosphatidylethanolamine; TG: Triglyceride

#### Lipid structure characteristics

**Fig. 3:**
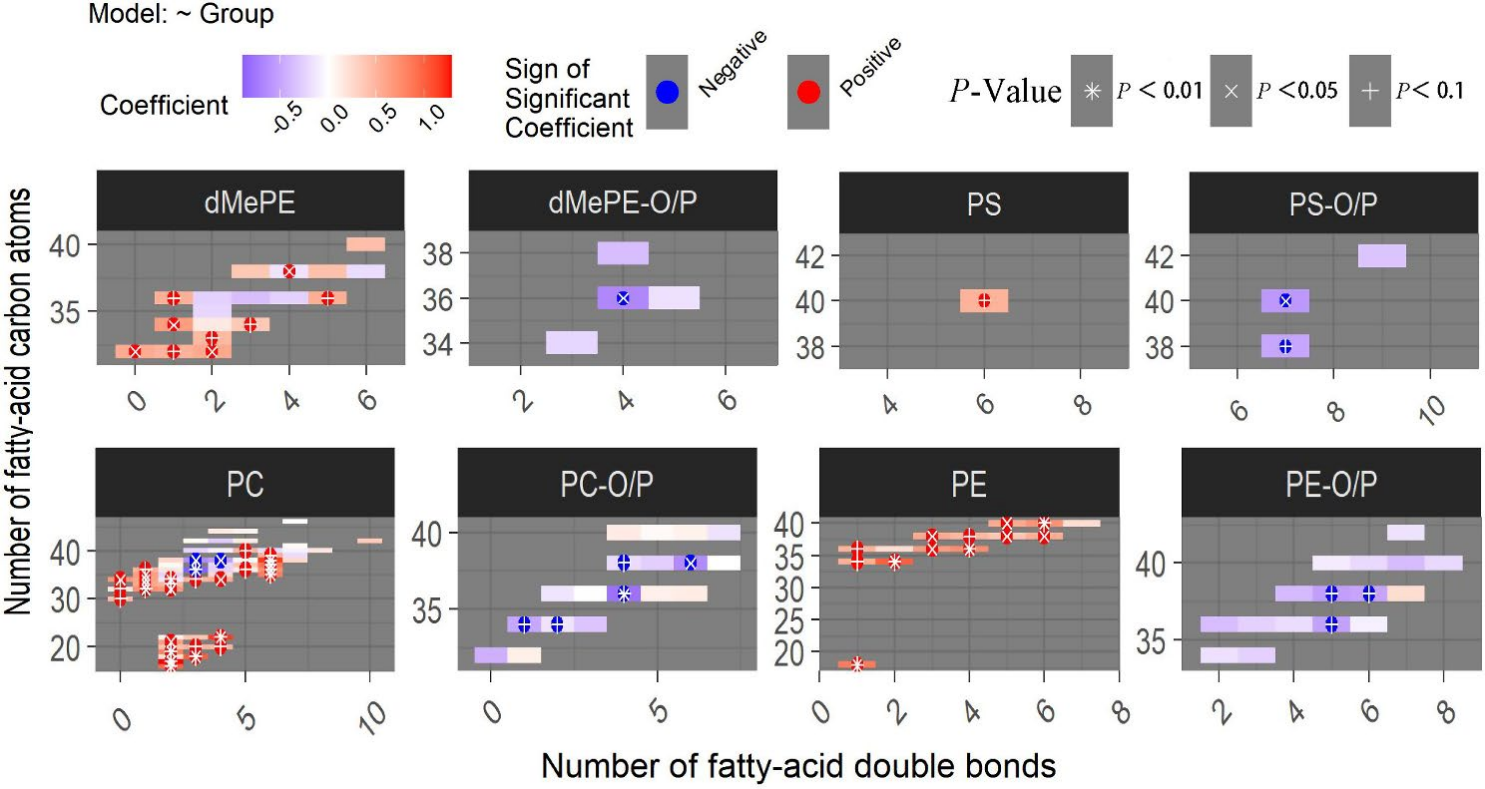
Heat map of the lipid structure characteristics. Individual lipid species were depicted by filled rectangles and arranged by lipids subclass according to the number of total carbon atoms (y axes) and number of double bonds (x axes). Color indicated the magnitude and direction of coefficient, and symbols indicated the *P*-value corresponded to the significance level of logistic regression models. Acronyms for lipids were shown in Fig. 2

Ether bonds at the sn-1 and ester bonds at the sn-2 sites are characteristics of plasmalogens. The analysis of plasmalogens, carbon atomic numbers, and carbon bond saturation levels were helpful to further understand the multimodal characteristics of lipid structure. In the AF-SSNHL group, PE O/P, dimethylphosphatidylethanolamine (dMePE) O/P, PC O/P, and phosphatidylserine (PS) O/P were downregulated, distinguishing them from most of PE, dMePE, PC, and PS (Fig. 3), which implied a reduction in antioxidative and anti-inflammatory factors.

### Lipidomic comparisons between responders and non-responders

#### Differential lipids identification

**Fig. 4:**
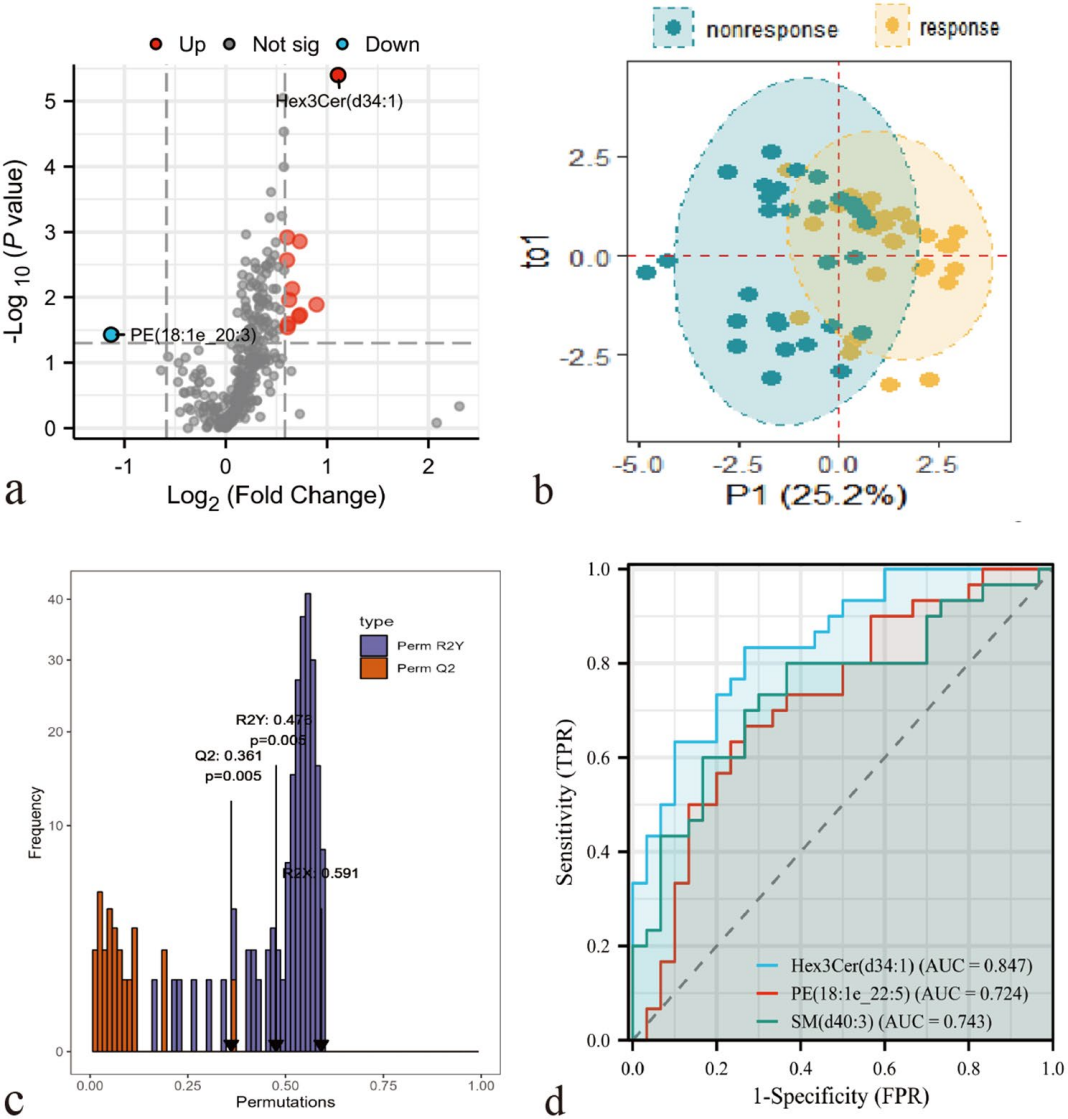
Differential lipids identification. (**a**) Volcano plot of the non-responders vs. responders. (**b**-**c**) Orthogonal partial least squares discriminant analysis (OPLS-DA): OPLS-DA model (**b**), and permutation test (**c**). (**d**) The area under the curve (AUC) of receiver operating characteristic curve (ROC) analysis. Acronyms for lipids were shown in Fig. 2

In total, 102 differentially expressed lipid molecules [43.14% SLs, 34.31% GPLs, 21.57% GPL-plasmalogens, and one monosialoganglioside 3(d34:1)] were identified in the responders vs. non-responders group (*P* < 0.05), of which 12 lipids met the FC > 1.5 or < 0.67 criteria (Fig. 4a and supplementary material 2). The OPLSDA model established with all lipid molecules was overfitting. Therefore, only 12 differential lipids were included to construct the OPLSDA model, which was found to partial distinguish between responders and non-responders (Fig. 4b). In permutation test, the R^2^Y = 0.476, Q^2^ = 0.361, which meaned that the degree of fitting and predictability were not high, but *P*-value < 0.01 (Fig. 4c). Ceramide (Cer)(d18:1_25:0), trihexosylceramide (Hex3Cer)(d34:1), PC(18:1e_22:5), PE(18:1e_22:5), and sphingomyelin (SM)(d40:3) had VIP values ≥ 1, in which Hex3Cer(d34:1), PE(18:1e_22:5), and SM(d40:3) met FDR-BH *P*-value < 0.05. Hex3Cer(d34:1) demonstrated optimal discriminative capacity with an area under the curve (AUC) of 84.7%, and sensitivity and specificity were 0.733 and 0.833, respectively (Fig. 4d).

#### Regression analysis

Hex3Cer(d34:1), PE(18:1e_22:5), SM(d40:3), and hearing loss were statistically significant in univariate regression (Table 3), so these factors were included in backward logistic regression to determine the extent of their impact on prognosis. It was found that Hex3Cer(d34:1) [OR (95% CI) = 5.708 (2.064–15.788), *P =* 0.001] and hearing loss [OR (95% CI) = 0.941 (0.897–0.986), *P* = 0.012] were independent determined factors. After adjusting for age, gender, anti-hypertensive therapy, lipid-lowering therapy, complications (tinnitus, vertigo, and stuffy ears), and the time to treatment, Hex3Cer(d34:1) and hearing loss still were significant predictors of prognosis (Table 3).

**Table 3:** Logistic regression model

Characteristics	FDR-BH *P*-value	FC value	VIP	Univariate logistic regression		Adjusted logistic regression	
				OR (CI 95%)	*P*-value	OR (CI 95%)	*P*-value
Hex3Cer(d34:1)	0.001	2.156	1.398	4.569 (1.817–11.488)	0.001	4.375 (1.595–11.997)	0.004
PE(18:1e_22:5)	0.043	1.658	1.025	2.242 (1.234–4.072)	0.008	2.591 (1.293–5.193)	0.007
SM(d40:3)	0.043	1.523	1.191	3.028 (1.374–6.670)	0.006	2.927 (1.227–6.983)	0.016
hearing loss				0.965 (0.936–0.996)	0.025	0.950 (0.906–0.996)	0.034

FDR-BH: Bonferroni and false discovery rate-benjamini hochberg; FC: Fold change; VIP: Variable importance in projection; OR: Odds Ratio; CI: Confidence Interval; Hex3Cer: Trihexosylceramide; SM: Sphingomyelin; PE: Phosphatidylethanolamine

#### Weighted gene co-expression network analysis

**Fig. 5:**
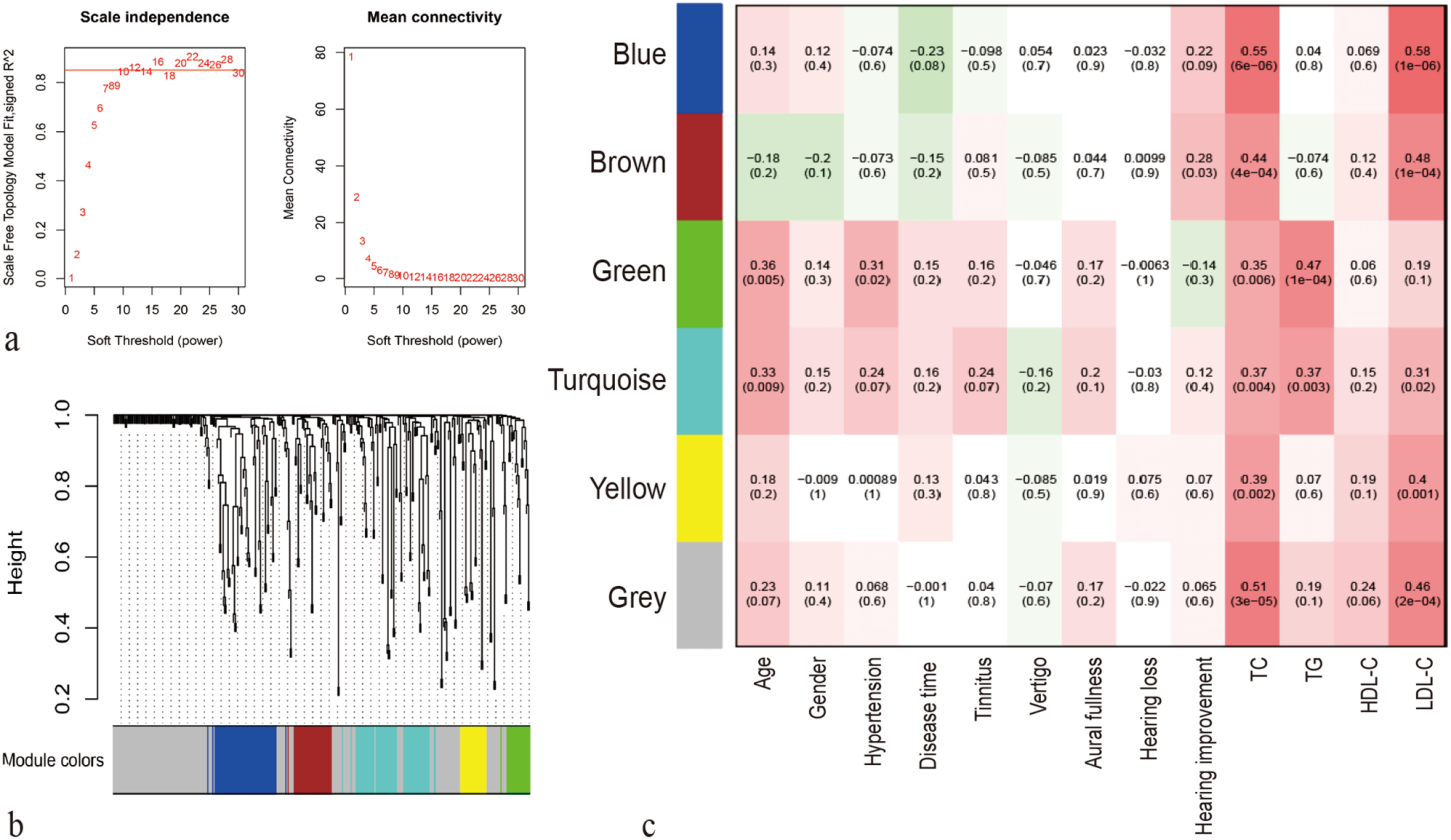
Weighted gene co-expression network analysis (WGCNA). The soft-thresholding powers selection of WGCNA (**a**), clustering dendrograms of the lipid species (**b**), a module-group relationship heatmap (**c**). Acronyms for lipids were shown in Fig. 2

WGCNA defined modules of highly associated lipids, correlating these modules with levels of hearing improvement. The WGCNA model’s R^2^ achieved 86.02% when the power value was 12, indicating a network pattern with scale-free (Fig. 5a). Subsequently, the expression matrix was transformed into a nearby matrix, and finally a topology matrix was obtained by further refinement. Lipids were clustered employing the hierarchical clustering technique of average-linkage. Six modules were obtained, and the grey module comprised lipids that could not be clustered into other modules (Fig. 5b). The blue, brown, green, yellow, and turquoise modules primarily comprised SM, GPL-plasmalogens, PE and TG, GPLs and Cer, and GPLs, respectively. Notably, a positive correlation emerged between the brown module and the level of hearing improvement (Fig. 5c).

### Correlation analyses

**Fig. 6:**
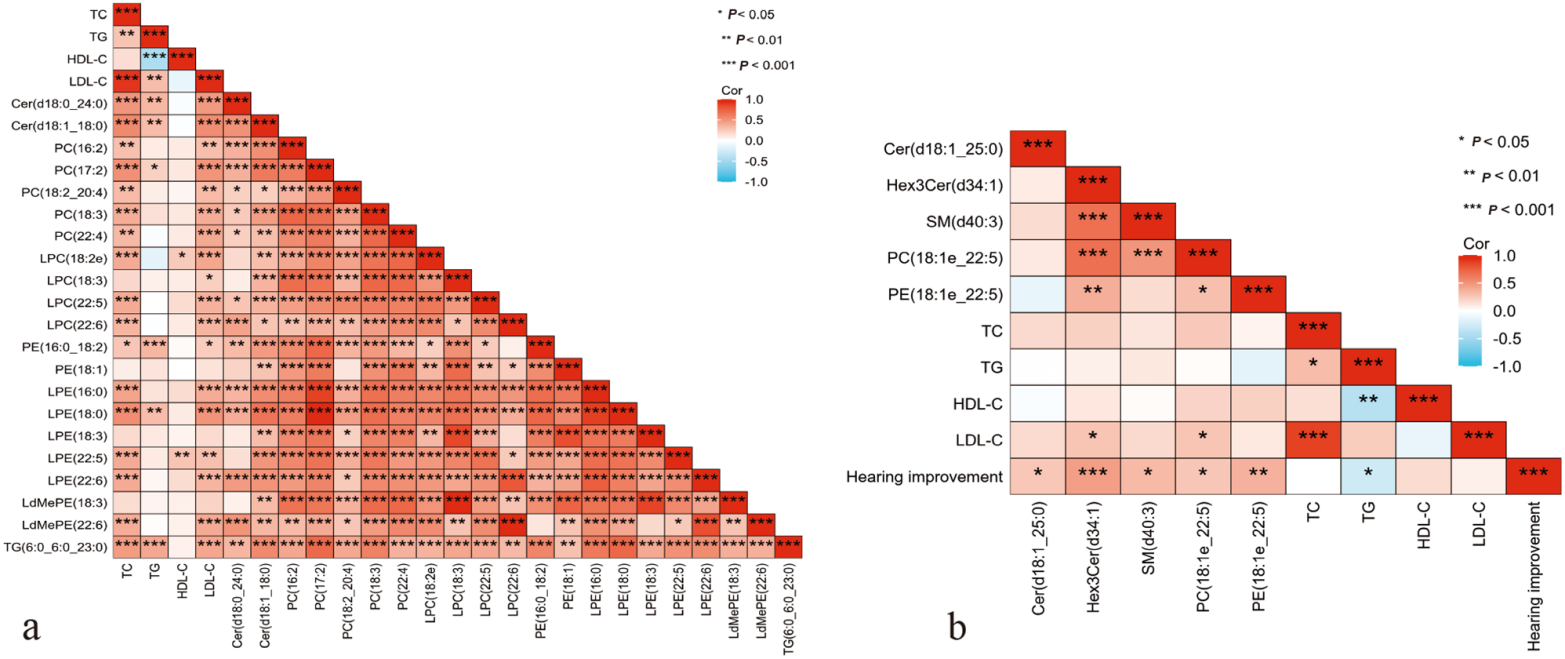
Spearman correlation analyses results. (**a**) Correlation heat maps depicting the level of total cholesterol (TC), triglycerides (TG), low-density lipoprotein cholesterol (LDL-C), high-density lipoprotein cholesterol (HDL-C), and differential lipid levels in the control vs. all frequencies sudden sensorineural hearing loss (AF-SSNHL) groups. (**b**) Correlation heat maps depicting the level of hearing improvement, TC, TG, HDL-C, LDL-C, and differential lipid levels in the non-responder vs. responder groups. Acronyms for lipids were shown in Fig. 2

The 85.71% of differential lipids between the control and AF-SSNHL groups positively correlated with the TC and LDL-C levels (Fig. 6a). The levels of hearing improvment significantly positively correlated with Cer(d18:1_25:0), Hex3Cer(d34:1), PC(18:1e_22:5), PE(18:1e_22:5), and SM(d40:3), but negatively correlated with TG (Fig. 6b).

## Discussion

A growing consensus has suggested that lifestyle plays a critical role in maintaining health, with long-term high-fat diets identified as a significant risk factor. In one study, mice with impaired glycolipid metabolism exhibited approximately 10 dB hearing loss at frequencies of 16 kHz and 32 kHz. Statins have been shown to rescue partial hearing loss and reduce the loss rate of outer hair cells in the cochlea basal turn [15]. The primary pathological changes in the cochlear tissue caused by hyperlipidemia included reduced vascular diameter and thickened vessel walls within the vascular stripe, and cell density was decreased in the spiral ganglion and spiral ligament located at the basal turn. [16]. Lipid metabolism was closely related to hearing function. This study was the first lipidomics investigation of AF-SSNHL, and found that the GLs, GPLs, and SLs levels increased in the AF-SSNHL group, particularly GPLs accompanied by metabolic disturbances. Besides, the differential lipids belonged to GPL-plasmalogens and SLs between responders and non-responderswere positively associated with the level of hearing improvement.

GPLs are the predominant phospholipids found in mammalian cell membranes and fulfill critical functions across diverse physiological processes, such as cell signaling, platelet activation, endoplasmic reticulum regulation, and mitochondrial stress management [17, 18]. Animal studies have suggested that the imbalance in PC and PE and their ratios might contribute to multiple metabolic disorders, such as endoplasmic reticulum stress, obesity, and insulin resistance [19–21]. In a prospective research carried out among Chinese residents of communities, PCs (16:0_18:1, 18:0_16:1, 18:1_20:3, 16:0_16:1, 16:0_20:3, 18:0_20:3), LPC (20:3), and PE (16:0_16:1) were positively associated with the development of diabetes over 6 years, in which partial GPLs correlated with an unhealthy diet and less exercise [22]. This author also supported the notion that PEs, particularly those composed mainly of C22:6, including PE (18:2_22:6), PE (18:1_22:6), PE (18:0_22:6), and PE (16:0_22:6) may raise the chance of developing metabolic syndrome [23]. Furthermore, PE (36:5) was found as a risk factor that contributed to cardiovascular disease during the 10-year follow-up [24]. Certainly, there were studies that expressed dissenting opinions, proposing that GPLs protected hearing [25–27]. For instance, lecithin has demonstrated protective properties against age-related hearing loss, safeguarding mitochondrial membrane potential and preventing oxidative damage to mitochondrial DNA [25]. Differential lipid molecules and their effects in different deafness diseases may vary, possibly owing to differences in the underlying pathogenic mechanisms, analysis platforms, and study populations.

Phospholipases or acyltransferases cleave the fatty acid chains of phospholipids, generating hydrophilic hydroxyl groups to form lysophospholipids. LPC acted as the pro-atherogenic component of oxidized low-density lipoprotein that triggered pro-inflammatory and pro-oxidative responses [28]. In patients with ischemic events, levels of LPE, LPI, and PE with mono-unsaturated or polyunsaturated fatty acid side chains were significantly higher than in individuals without such events. Moreover, patients displaying elevated levels of LPE showed increased platelet aggregation in ex vivo conditions. The results indicated that LPE may promote platelet hyperreactivity, potentially leading to a greater chance of thromboembolic complications in those with coronary artery disease [29]. Both studies indicated that LPC and LPE were risk factors for vascular injury or ischemic tissue damage.

Plasmalogens, which are synthesized in peroxisomes, are a distinct class of GPLs that are localized to cellular membranes, organelles, and lipid rafts. They serve not merely as structural membrane components, but also as lipid-derived second messengers, playing pivotal roles in the retention of long-chain polyunsaturated fatty acids, cholesterol efflux, and ion transport [30]. Plasmalogens, with their vinyl ether bonds, acted as exceptionally effective antioxidants against reactive oxygen species. PE-plasmalogens, known for their role in protecting endothelial cells from oxidative damage without inducing excessive toxic oxidation [31]. Interestingly, in the present study, partially corresponding plasmalogens exhibited the opposite trend with PE, dMePE, PC, and PS between the control and AF-SSNHL groups. In addition, PE (18:1e_22:5) was identified as a significant prognostic predictor, even after adjusting for age.

The lipid rafts found in the plasma membrane of cochlear cells were enriched with SLs, glycosphingolipids, gangliosides, and cholesterol and contained calcium ion channels and calcium-sensitive potassium ion channels. Therefore, it may involve a wide range of auditory processes, including cochlear mechanics and sensory transduction [32]. Cer constitutes the pivotal molecule within sphingolipid metabolism. Through the catalytic action of sphingomyelin synthase (SMS), Cer is converted into SM. And the incorporation of one or more glycosyl onto Cer by specific glycosyltransferases yields HexCer. Subsequent enzymatic glycosylation processes can further elaborate these structures into complex glycosphingolipids such as gangliosides and galactocerebrosides. Cer can be catabolized into sphingosine and free fatty acids, with sphingosine undergoing phosphorylation by sphingosine kinase to yield sphingosine-1-phosphate (S1P) [33]. Gene knockout mice targeting the S1P receptor, and SMS-1 have demonstrated varying degrees of hearing loss, accompanied by atrophy of the stria vascularis, disorganization of marginal cells, and decreased cochlear potentials [34, 35]. SLs metabolites modulated the apoptotic response of hair cells to gentamicin-induced toxicity. While Cer intensified the ototoxic effects by facilitating apoptosis, S1P and gangliosides served as protective agents for the cochlea [36]. HexCers exhibited pro-proliferative and anti-apoptotic properties, potentially through modulation of the intracellular ceramide reservoir [37].

### Strengths and limitations

This study utilized high-throughput untargeted lipidomics to explore the changes in over 1,000 lipid molecules of patients with AF-SSNHL. Some differentially expressed TG lipid molecules were screened despite no appreciable variations in clinical TG level between the AF-SSNHL and control groups, highlighting the high sensitivity of lipometabolomics to lipid changes and the advantage of identifying early metabolic abnormalities. This study proposed that dyslipidemia may serve as the underlying mechanism for AF-SSNHL pathogenesis, and the aberrant metabolism of SLs and GPL-plasmalogens were closely associated with prognosis. The question of whether rectifying abnormal lipid metabolism can prevent and ameliorate AF-SSNHL remains a subject that necessitates further investigation and resolution in the future. Nonetheless, this study had some limitations: First, blood samples from patients with AF-SSNHL were collected within 2 weeks after onset; therefore, long time windows may result in lipid metabolism changes due to diet or lifestyle alterations. Second, it did not directly reflect cochlear lipid metabolism.

## Conclusion

The patients with sudden sensorineural hearing loss across all frequencies exhibited elevated levels of glycerolipids, glycerophospholipids, and sphingolipids, and pronounced glycerophospholipids metabolic abnormalities, which may have contributed to the pathogenesis. Sphingolipids and plasmalogens were associated with the level of hearing improvement. By understanding the lipid profile changes, clinicians may be able to predict the prognosis of hearing recovery and personalize treatment approaches.

### Electronic supplementary material

Below is the link to the electronic supplementary material.

Supplementary Material 1

Supplementary Material 2

## Data Availability

The datasets are available from the corresponding author on reasonable request.

## Abbreviations

SSNHL: Sudden sensorineural hearing loss
AF-SSNHL: All frequencies-SSNHL
TC: Total cholesterol
m/z: mass-to-charge ratios
FC: Fold change
OPLS-DA: Orthogonal partial least squares discriminant analysis
VIP: Variable importance in projection
OR: Odds ratios
CI: Confidence intervals
ROC: Receiver operating characteristic
AUC: Area under the ROC curve
WGCNA: Weighted gene co-expression network analysis
LDL-C: Low-density lipoprotein cholesterol
TG: Triglyceride
GLs: Glycerolipids
GPLs: Glycerophospholipids
SLs: Sphingolipids
LPC: Lysophosphatidylcholine
PC: Phosphatidylcholine
MePC: Methylphosphatidylcholine
LPE: Lysophosphatidylethanolamine
PE: Phosphatidylethanolamine
LdMePE: Lysodimethylphosphatidylethanolamine
dMePE: Dimethylphosphatidylethanolamine
PS: Phosphatidylserine
Cer: Ceramide
Hex3Cer: Trihexosylceramide
SM: Sphingomyelin
HDL-C: High-density lipoprotein cholesterol
