# Supplementary material for: Serum lipid metabolism characteristics and potential biomarkers in patients with unilateral sudden sensorineural hearing loss
Source: Lipids Health Dis. 2024 Jun 29;23:205. doi: 10.1186/s12944-024-02189-8 (PMC11218322; doi:10.1186/s12944-024-02189-8)
Supplement: Supplementary file 1 — Supplementary Material 1 [file 12944_2024_2189_MOESM1_ESM.docx]

**1.1 Metabolite extraction**

Thawed serum samples were placed on dry ice to prepare for liquid chromatography-mass spectrometry (LC-MS). First, 100 µL of the sample was mixed with 300 µL of methanol, then vortexed for 2 min at 4°C and 2,000 rpm, followed by sonication in an ice bath for 5 minutes. Then, 1,000 µL of methyl tert-butyl ether was added, and the mixture was vortexed for 2 min at 4°C and 2,000 rpm, followed by incubation at 4°C for 30 min. Next, 300 µL of water was added, and the mixture was vortexed for 2 min at 4°C and 2,000 rpm, followed by incubation at 4°C for 10 min. The sample was centrifuged at 4°C and 12,000 rpm for 15 min, and an equal volume of the supernatant was transferred to an Eppendorf tube for vacuum drying. For reconstitution, 100 µL of an isopropanol (IPA): acetonitrile (ACN) solution (9:1) was added to the dried sample, vortexed for 30 s, and sonicated in an ice bath for 5 min. The reconstituted sample was centrifuged at 4°C and 12,000 rpm for 10 min. Finally, 20 µL of supernatant was taken into the sample vial and analyzed by the instrument. Quality control (QC) samples were created by mixing 10 µL of supernatant from each sample, which were used to assess the instrument’s status and ensure a balanced LC-MS system.

**1.2 Chromatographic separation and mass spectrometry acquisition**

Samples were placed in an 8°C autosampler, and the components in the samples were separated using an ACQUITYTM Premier CSH C18 chromatographic column (volume: 2 µL; column temperature: 50°C; flow rate: 0.3 mL/min). Chromatographic mobile phase A: 3:2 ratio of ACN/H2O (10 mM ammonium formate), and chromatographic mobile phase B: 9:1 ratio of IPA/ACN (10 mM ammonium formate). Following separation by LC-MS, the samples were analyzed using a Q Exactive Plus mass spectrometer (Thermo Fisher Scientific, Waltham, MA, USA) equipped with a dual electrospray ionization (ESI) source. Ionization was performed in either positive ion mode (ESI+) or negative ion mode (ESI–). The temperature of the capillary tube and the auxiliary heater were set at 325°C and 300°C, respectively; the sheath gas flow rate was 30 a.u.; the auxiliary gas flow rate was 10 arb; and the spray voltage was 2.5 kV (+)/2.5 kV (–). The S-Lens RF was set at 50 a.u. The secondary mass spectrum collision energy was set to 30, and ten priority scans were performed (Top N=10). The mass range of the mass spectrometry was from 200 to 1800 m/z, and positive and negative ions were detected separately.

**1.3 Quality control**

To ensure the data set's high quality and more accurate lipid detection, quality assurance procedures were frequently implemented. Principal component analysis was used as an unsupervised data analysis method to examine group trends and identify outliers. The QC samples clustered tightly, indicating good reproducibility, and no extreme outliers were observed (Suppl. Fig. 1a). In addition, the relative standard deviation (RSD) of each characteristic peak in the QC samples, which is the coefficient of variation, was calculated. Characteristic peaks with an RSD greater than 30% should be removed from the analysis because they were not reproducible and could affect the confidence of the results (Suppl. Fig. 1b). In total, 37 lipid subclasses were identified, including 630 lipid molecules in cationic mode (Suppl. Fig. 1c) and 559 lipid molecules in anionic mode (Suppl. Fig. 1d).


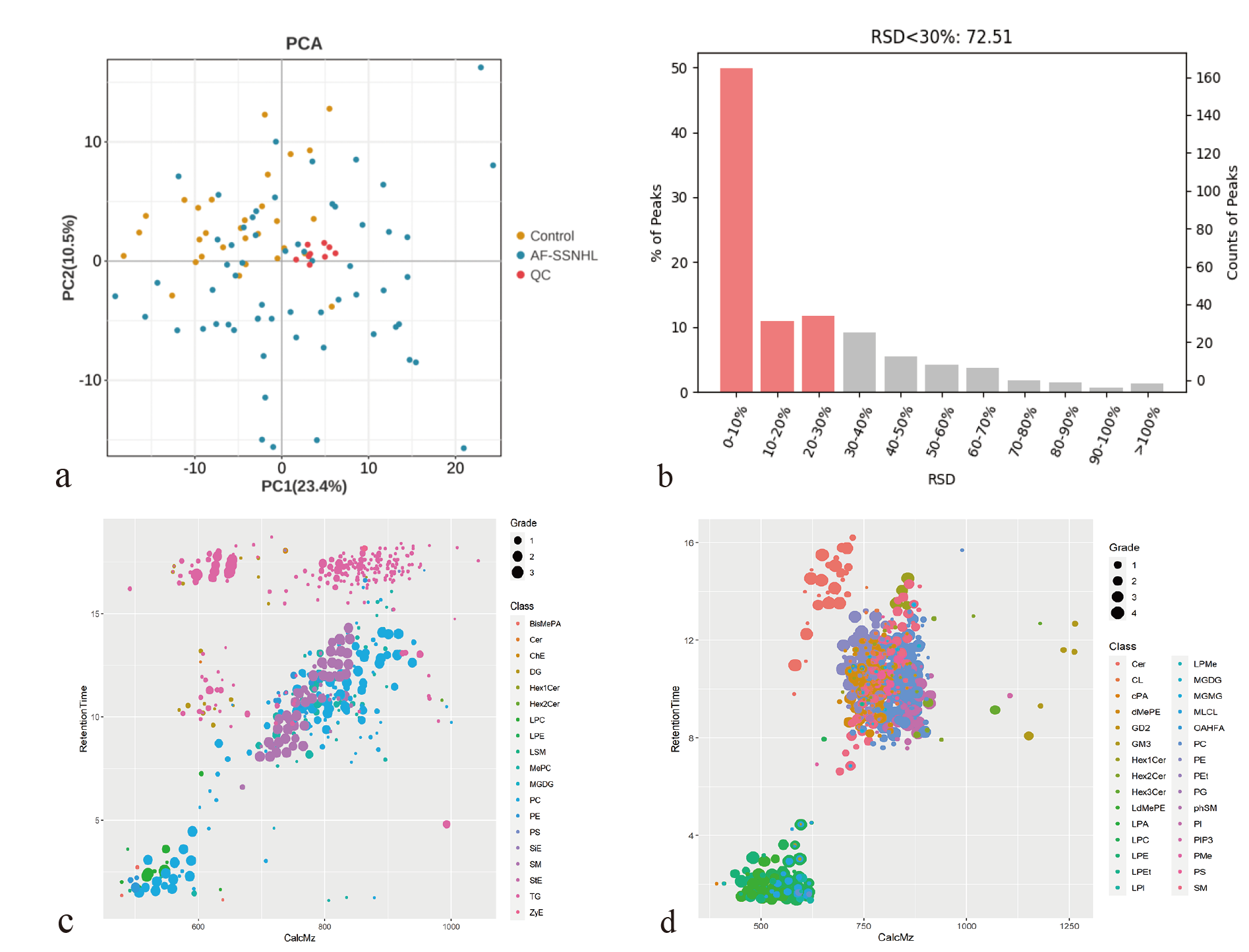


Suppl. Fig. 1: Quality control plot and lipidomic profile. (a) Principal component analysis, where the dots of brown, blue, red represent the control group, the AF-SSNHL group, and quality control (QC) samples, respectively. (b) Distribution plot of the relative standard deviation, showing that 72.51% of characteristic peaks in QC samples had an RSD less than 30%. Lipidomic profile analysis. (c, d) Scatter plots of lipid subclasses in the (a) cationic and (b) anionic modes. BisMePA: Bis-methyl phosphatidic acid, Cer: Ceramide, ChE: Cholesterol Ester, DG: Diacylglycerol, Hex1Cer: Monohexosylceramide, Hex2Cer: Dihexosylceramide, LPC: Lysophosphatidylcholine, LPE: Lysophosphatidylethanolamine, LSM: Lysosphingomyelin, MePC: Methylphosphatidylcholine, MGDG: Monogalactosyldiacylglycerol, PC: Phosphatidylcholine, PE: Phosphatidylethanolamine, PS: Phosphatidylserine, SiE: Sitosteryl ester, SM: Sphingomyelin, StE: Sterol Ester, TG: Triacylglycerol, ZyE: Zymosterol Ester, CL: Cardiolipin, cPA: Cyclic Phosphatidic Acid, dMePE: Dimethylphosphatidylethanolamine, GD2: Disialoganglioside, GM3: Monosialoganglioside, Hex3Cer: Trihexosylceramide, LdMePE: Lysodimethylphosphatidylethanolamine, LPA: Lysophosphatidic Acid, LPEt: Lysophosphatidylethanol, LPI: Lysophosphatidylinositol, LPMe: Lysophosphatidylmethanol, MGMG: Monogalactosylmonoacylglycerol; MLCL: Monolysocardiolipin, OAHFA: (O-acyl)-1-hydroxy fatty acid, PEt: Phosphatidylethanol, PG: Phosphatidylglycerol, phSM: Phytosphingosine-containing Sphingomyelin, PI: Phosphatidylinositol, PIP3: Phosphatidylinositol-trisphosphate, PMe: Phosphatidylmethanol.
